# How loneliness mediates the association between migration status and health trajectories: Longitudinal evidence from Germany

**DOI:** 10.1016/j.jmh.2026.100408

**Published:** 2026-03-30

**Authors:** Songyun Shi, Silvia Loi

**Affiliations:** a Max Planck Institute for Demographic Research, Rostock, Germany; b School of Geography, University College Dublin, Dublin, Ireland; c Max Planck – University of Helsinki Center for Social Inequalities in Population Health, Rostock, Germany and Helsinki, Finland

**Keywords:** Germany, Loneliness, Longitudinal analysis, Mediation analysis, Mental health, Migrant health

## Abstract

**Objectives:**

Evidence shows that immigrants tend to have better health upon arrival, but they experience faster health deterioration than non-immigrants. However, rarely do we assess the role of loneliness in shaping health disparities by migration status. This study examines how loneliness mediates the relationship between migration status and health.

**Methods:**

Using nationally representative data from the 2012–2020 German Socio-Economic Panel, we apply a parallel process latent growth curve model with mediation analysis to examine the direct associations of migration status with mental and physical health trajectories, as well as the mediating role of loneliness, by gender and age at migration (6609 non-immigrants; 634 immigrants).

**Results:**

Among women loneliness mediates the relationship between migration status and mental health (*β* = −0.013, 95 % CI = −0.022 - −0.006, *p* < 0.01). Immigrants are more likely to experience loneliness, which is associated with poorer mental health. Analyses that account for gender and age at migration differences reveal that loneliness mediates the relationship only among women who moved to Germany after age 18 (*β* = −0.015, 95 % CI = −0.024 - −0.008, *p* < 0.001).

**Discussion:**

Loneliness contributes to mental health disparities between immigrant and non-immigrant women. Immigrant women who migrated after age 18 are particularly vulnerable. We do not observe the same mechanism among men, nor for physical health. Given that the mechanisms vary by age at migration and gender, future studies should tailor their analyses to specific population subgroups to better understand the drivers of migration-related health disparities.

## Highlights

• Loneliness explains mental health disparities between immigrants and non-immigrants.
• The mediating effect of loneliness on mental health is only significant among women.
• Loneliness contributes to mental health disparities among immigrants who migrate at older ages.
• Women who migrated after age 18 are particularly vulnerable to loneliness and poor mental health.
• This study introduces parallel process latent growth curve modelling in migrant health studies.

## 1 Introduction

Although immigrants tend to have better health upon arrival, their health deteriorates more rapidly than that of non-immigrants, often resulting in similar or even worse health outcomes in later life (Antecol and Bedard, 2006; Loi et al., 2024, 2025; Nesterko et al., 2019). Previous studies document health disparities by migration status, and often explain these disparities by focusing on behavioral or social structural factors, such as diet, material deprivation, and access to health care (Akresh, 2007; Ferrara et al., 2024; Loi and Hale, 2019). However, socio-psychological mechanisms such as loneliness remain unexplored.

Loneliness has become a public health concern and is associated with a range of adverse mental and physical health outcomes (Hawkley and Cacioppo, 2010; Park et al., 2020). Evidence also shows that immigrants are more likely to report higher levels of loneliness (De Witte and Van Regenmortel, 2022; Delaruelle, 2023; Fokkema and Naderi, 2013). However, rarely do we assess how socio-psychological mechanisms such as loneliness may contribute to explaining health disparities between immigrants and non-immigrants, especially using longitudinal designs.

To address this knowledge gap, this study investigates whether loneliness contributes to explaining the mental and physical health disparities between immigrants and non-immigrants. We specifically focus on loneliness, as previous literature has demonstrated that immigrants face barriers in rebuilding their social networks in the receiving country after migration, and are often at heightened risk of experiencing loneliness, which in turn is linked to adverse health outcomes (Hurtado-de-Mendoza et al., 2014).

Using a longitudinal panel survey in Germany, we apply mediation analysis to examine whether and how loneliness contributes to mental and physical health disparities between immigrants and non-immigrants, and to assess the role of gender and age at migration in this relationship. We find that loneliness contributes to mental health disparities by migration status, but only among women and those who migrated at older ages.

### 1.1 Immigrant health

Immigrants often have better health than non-immigrants upon their arrival in the receiving country, due to a phenomenon known as the healthy immigrant effect (Markides and Rote, 2018). This phenomenon is considered a paradox given immigrants’ lower socioeconomic position than non-immigrants, which would be expected to be associated with poorer health. At least three hypotheses have been proposed to explain the immigrant health advantage. First, the selectivity hypothesis suggests that immigrants are positively selected in their country of origin, meaning their pre-migration factors may contribute to better health upon arrival (Feliciano, 2020). Second, the cultural hypothesis asserts that immigrants engage in more favorable health behaviors compared with non-immigrants, which positively impacts their health (Riosmena et al., 2017). Third, the “salmon bias,” which suggests that older immigrants with poor health tend to return to their country of origin, resulting in an artificial overestimation of good health among immigrants who stay in the receiving countries (Palloni and Arias, 2004).

However, despite this health advantage, immigrants’ health deteriorates more rapidly over time than that of non-immigrants, leading to a diminished health advantage or even worse health outcomes later in life. This pattern, often referred to as unhealthy assimilation, has been observed across multiple countries, such as the United States, Italy, and Germany (Antecol and Bedard, 2006; Ferrara et al., 2024; Loi and Hale, 2019; Loi et al., 2025). Previous studies attribute the immigrants’ health decline to structural factors, neighborhood environment, and cultural assimilation. Structural explanations include discrimination (Agudelo-Suárez et al., 2011), limited health care access (Antecol and Bedard, 2006), greater vulnerability to adverse life events (Loi et al., 2024), and poor economic and social conditions (Loi and Hale, 2019). The literature on neighborhood effects suggests that immigrants are less able to relocate to more resourceful neighborhoods, resulting in greater exposure to disadvantaged neighborhood environments (Lersch, 2013). Cultural assimilation, including adopting the drinking or smoking behaviors of the receiving country, may also be responsible for gradually diminishing immigrants’ initial health advantage (Akresh, 2007).

The majority of studies in Europe show a similar pattern for immigrants’ mental health: immigrants tend to have better mental health upon arrival, but their mental health levels converge to, or even fall below, those of non-immigrants over time (e.g., Brunori, 2024; Ferrara et al., 2024; Holz, 2022). However, the mechanisms behind this phenomenon remain unclear. For instance, it is unclear whether the convergence is driven by individual-level health decline or by compositional factors such as immigrant cohort differences or selective return migration. Evidence from Germany shows a faster individual-level decline in immigrants’ mental health, whereas no such evidence has been found in the United Kingdom (Brunori, 2024; Ferrara et al., 2024).

Migration-specific factors, including age at migration and length of stay in the receiving country, have been found to modify immigrants’ health risks. Migrating at an older age often implies lower levels of social integration, as immigrants encounter greater barriers to entering the receiving country, compared to those who migrated during childhood. Meanwhile, longer duration of stay may reflect prolonged exposure to adverse environments in the receiving country, which can contribute to poorer health outcomes (Lanari and Bussini, 2012; Loi and Hale, 2019). Similar patterns are observed in mental health, with age at migration and length of stay linked to poorer mental health (Honkaniemi et al., 2020).

### 1.2 Loneliness as a mediator

Loneliness can be understood as a lack of integration into a personal network, characterized by an absence of closeness, intimacy, and a sense of meaning in life (van Tilburg, 2020). The association between loneliness and adverse physical and mental health outcomes across multiple contexts has been well documented (Park et al., 2020). The loneliness model suggests that lonely individuals feel less safe and perceive greater social threat than non-lonely individuals. Lonely individuals tend to expect negative social interactions, and may behave in ways that elicit confirming responses from others, creating a self-fulfilling cycle. This self-reinforcing loop of loneliness is accompanied by hostility, stress, pessimism, anxiety, and low self-esteem, and activates neurobiological and behavioral mechanisms that contribute to adverse health outcomes. In sum, loneliness can affect health through behaviors (e.g., sleep) and biological factors (e.g., neuroendocrine responses) (Hawkley and Cacioppo, 2010). Given this evidence, loneliness has been recognized as a public health issue and as such is receiving increased attention (Hawkley, 2022).

Immigrants are often seen as more vulnerable to loneliness because of various migration-specific risk factors, such as limited proficiency in the receiving country’s language, which prevents them from being fully included in the receiving society, experiences of discrimination, a weak sense of belonging, and cultural conflicts (Delaruelle, 2023). In fact, previous evidence clearly shows that immigrants are more likely to report higher levels of loneliness (De Witte and Van Regenmortel, 2022; Fokkema and Naderi, 2013; Joshi et al., 2024; Wu and Penning, 2015). Migration-related factors can also influence immigrants’ loneliness levels. Migrating at older ages is an important risk factor, as older immigrants are more likely to face challenges learning a new language, and building social networks. These difficulties hinder integration, resulting in higher loneliness rates, and in turn, poorer health outcomes (Delaruelle, 2023; Treas and Batalova, 2009; Treas and Mazumdar, 2002).

### 1.3 Gender disparities

Previous literature documents gender differences in health disparities by migration status. Physical health disparities between immigrant and non-immigrant women are greater than those between men (Loi et al., 2025; Trappolini and Giudici, 2021). Moreover, immigrant women are more likely to report poorer mental health than immigrant men (Potochnick and Perreira, 2010). These findings suggest that immigrant women are particularly vulnerable to both physical and mental health. They face double vulnerability as immigrants and as women, which may lead to poorer health outcomes (Llácer et al., 2007).

Moreover, the impact of loneliness on health appears to vary by gender, with loneliness having a greater effect on women’s health (Boehlen et al., 2023, 2022). However, evidence on gender differences in loneliness is mixed. Some studies find no gender differences (Maes et al., 2019), others report higher levels among women (Delaruelle, 2023), and still others show the opposite pattern (Fokkema and Naderi, 2013). Less is known about loneliness among immigrant men and women, or about how the mediating effect of loneliness may vary by gender and migration.

## 2 Aims and research questions

This study is the first to examine the mediating role of loneliness in shaping health disparities by migration status in the European context, and applies an innovative approach, useful in longitudinal settings. This study addresses several limitations in prior research. First, most existing studies focus on describing health disparities rather than examining their underlying mechanisms. Second, previous explanations for health disparities across migration status have largely emphasized socio-economic, behavioral and structural factors (e.g., Loi and Hale, 2019), while few studies have considered socio-psychological factors (e.g., loneliness), as potential contributors. Finally, most studies use cross-sectional designs (e.g., Delaruelle, 2023), which limits their ability to capture dynamic health changes or uncover the mechanisms behind health disparities.

We employ an innovative approach, the parallel process latent growth curve model (PPM) with mediation analysis, to examine whether loneliness contributes to health disparities between immigrants and non-immigrants, and whether this association varies by gender. This approach allows us to model multiple health outcomes simultaneously, distinguish between individual differences and within-individual change, and directly examine loneliness as a mediator of health across migration status (Muthen, 2004). In addition, we investigate whether the mediating role of loneliness differs depending on the age at migration.

First, we employ a PPM with mediation analysis to investigate whether loneliness mediates the relationship between migration status and mental and physical health trajectories. The analysis is stratified by gender. Next, we apply the same analytical approach to examine whether the mediating association of loneliness between migration status and health trajectories varies by age at migration and gender. The two sets of hypotheses are listed below.

H1.1 Loneliness mediates the association between migration status and mental and physical health trajectories.

H1.2 This mediating relationship (H1.1) varies by gender.

H2.1 The mediating role of loneliness in mental and physical health trajectories varies by age at migration, with those who migrate at older ages showing higher levels of loneliness and poorer health.

H2.2 This mediating relationship (H2.1) varies by gender.

## 3 Methods

### 3.1 Data and sample

We use data from the German Socio-Economic Panel (G-SOEP), a yearly nationally representative panel survey in Germany that started in 1984 (Goebel, 2023). The survey includes ∼30,000 individuals in 15,000 households, covering information on family structure, occupation, education, income, health, and well-being. To reduce language-related selection bias among immigrants, the G-SOEP offers questionnaires in Italian, Spanish, Turkish, and other languages (Gauer and Kristen, 2023).

**Fig. 1:**
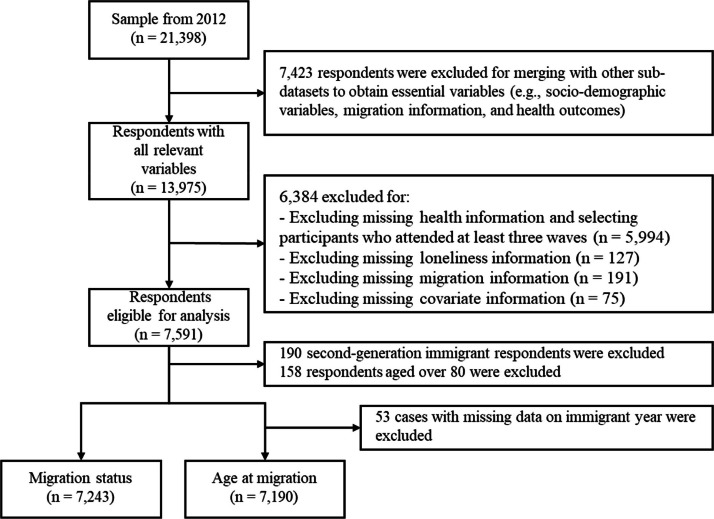
Sample selection procedure.

We restrict the G-SOEP core sample to 2012–2020, as loneliness was first measured in 2013. To address potential reverse causality effect, whereby individuals with poor health are more likely to experience loneliness, we used lagged controls that account for mental and physical health status in 2012 (Wickrama et al., 2016). Since mental and physical health data are collected biennially, we include waves 2012, 2014, 2016, 2018, and 2020. We select individuals who participated in at least three waves from 2014 to 2020, and restrict the sample to individuals under age 80. Although excluding individuals aged over 80 may reduce generalizability, it helps control for the “salmon bias” and prevents overestimation of immigrants’ health. The final analytical sample for investigating differences in loneliness by migration status consists of 7243 individuals, while the sample for examining variation by age at migration includes 7190 individuals (53 cases are missing data on the year of immigration). See Fig. 1 for the detailed sample selection procedure.

### 3.2 Measures

Our outcomes are the physical (PCS) and the mental component summary (MCS), measured in 2014, 2016, 2018, and 2020. The two outcomes are measured biennially since 2002 based on the SOEP-specific version of the 12-item Short-Form Health survey (SF-12v2). The SF-12v2 is a frequently used and reliable measure of overall health status (Ware et al., 2002). The PCS and MCS variables are computed through exploratory factor analysis and converted into standardized 0–100 scores, with higher values corresponding to better health (Andersen et al., 2007). These variables have been used in previous studies to assess physical and mental health (Ferrara et al., 2024; Nesterko et al., 2019). We use the PCS and MCS scores to assess mental and physical health trajectories.

The mediator in this study is loneliness measured in 2013. We use a single time point for loneliness due to dataset limitations: it was not measured every two years like the health outcomes, and we have only one measurement of loneliness prior to the baseline health outcomes (2014). Loneliness is measured using the three-item short version of the UCLA Loneliness Scale, which asks respondents how often they feel (1) a lack of companionship, (2) left out, and (3) isolated from others (Hughes et al., 2004). Each item is rated on a five-point scale, ranging from “never” to “very often.” We calculate the mean score of the three items, resulting in a scale ranging from 1 to 5, with higher scores indicating greater loneliness. This measure demonstrates good validity and internal consistency in the analytical sample (Cronbach’s α = 0.77) (Russell, 1996). Details on the scale’s development and validation can be found in Hughes et al. (2004).

The main predictors are migration-related factors, including migration status and age at migration. We define migration status using the place of birth criterion: an individual is an immigrant if s/he was born outside of Germany, and is a non-immigrant if s/he was born in Germany (0 = non-immigrant; 1 = immigrant). We calculate age at migration by subtracting the birth year from the immigrant year (age at migration = immigrant year – birth year). Age at migration is categorized into two groups (1 = 0–17 years; 2 = 18+ years) to capture the varying impacts of migration at different life stages, with non-immigrants serving as the reference category (0 = non-immigrant). These cut-offs balance sample size considerations and reflect critical life-course periods, which may have distinct long-term effects on health (Gubernskaya, 2014; Honkaniemi et al., 2020). We also note that the length of stay in Germany is an important indicator. However, among men, the sample size for immigrants with <10 years of residence is below 30, reducing statistical power. Therefore, we include the analyses of the length of stay only in the sensitivity analysis.

The socio-demographic covariates include age (18–80), gender (0 = women; 1 = men), educational attainment (1 = less than high school; 2 = high school; 3 = more than high school), household income quartiles (1 = lowest 25 %; 2 = 25–50 %; 3 = 50–75 %; 4 = highest 25 %), household size (1–10), marital status (0 = other; 1 = married), and employment status (0 = unemployed; 1 = employed). These covariates were drawn from the baseline of the study (G-SOEP 2012). To account for potential reverse effects from health to loneliness, we include mental and physical health status in 2012 (baseline). We also create a binary attrition variable to model health missingness over time (0 = participation in all four waves; 1 = non-participation in all four waves).

### 3.3 Analytical strategy

**Fig. 2:**
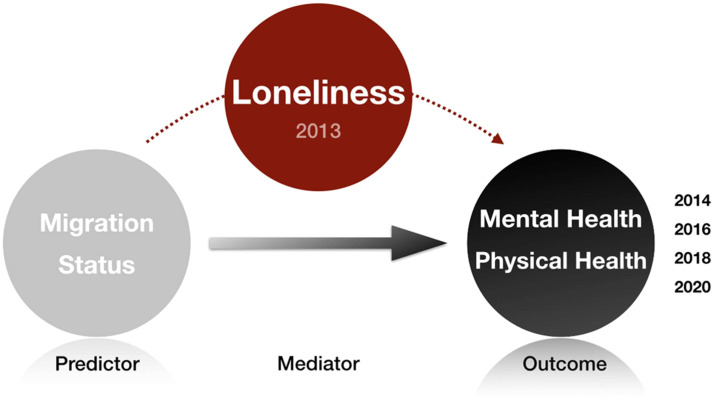
Conceptual model of the mediation analysis from migration status to health through loneliness.

Our statistical analysis consists of three main steps. First, we use descriptive and bivariate analyses to explore the distribution of key variables and examine differences between immigrants and non-immigrants by gender using χ² tests and Welch *t*-tests. This analysis is conducted using Stata 18. Next, we use a PPM with mediation analysis to examine the direct associations of migration-related factors (migration status and age at migration) with mental and physical health trajectories (initial health status [i.e., intercept] and longitudinal changes [i.e., slope]), as well as their indirect associations through loneliness. In order to design a mediation model, we created a time lag between predictors, mediators and outcomes. Predictors were collected in 2012, loneliness was measured in 2013, and health outcomes were measured in 2014, 2016, 2018 and 2020 (see Fig. 2). These analyses are then stratified by gender to examine gender differences in these relationships. We chose the stratified approach because it allows all parameters to vary freely across groups and produces group-specific indirect associations that are clearer to interpret, aligning with our research objectives.

The PPM is conducted sequentially. We first estimate two unconditional latent growth curve models (LGCMs) for mental and physical health to identify baseline health status and longitudinal changes in health outcomes. We then combine these models into a parallel process model to analyze dual health trajectories. Third, the parallel process model is conditioned on migration status, loneliness, and covariates to examine both the direct associations of migration status with mental and physical health trajectories and their indirect associations through loneliness. The analysis is then stratified by gender. Finally, we apply the same model to assess associations between age at migration and health trajectories and their indirect associations through loneliness, with results stratified by gender.

We perform LCGM and PPM with mediation analysis using M*plus* 8.11 and handle missing values using full information maximum likelihood (FIML) to include more cases in the analysis (Enders and Bandalos, 2001; Muthén & Muthén, 1998–2017; Wickrama et al., 2016). We use the following metrics to assess model fit: model χ^2^, the comparative fit index (CFI), the Tucker-Lewis Index (TLI), the root mean square error of approximation (RMSEA), and the standardized root mean square residual (SRMR). The recommended cut-offs for a proper model are CFI and TLI above 0.9 and RMSEA and SRMR below 0.08 (Hu and Bentler, 1999). The mediation effect is evaluated using the delta method, along with 1000 bootstrapped replications to get the bootstrapped confidence intervals (Muthén & Muthén, 1998-2017).

## 4 Results

### 4.1 Descriptive statistics and bivariate analysis

Descriptive statistics of the full sample and bivariate analysis for migration status by gender are presented in Table 1. Mental health levels for the full sample remained relatively stable over time, while physical health showed a declining trend, with the mean score decreasing from 49.18 (SD = 9.75) in 2012 to 47.15 (SD = 10.26) in 2020. The average loneliness score for the full sample was 1.96 (SD = 0.72). Of the sample, 8.75 % were immigrants, with 68.14 % having migrated to Germany after age 18. The average age of the full sample was 53.19 years (SD = 14.65). Over 90 % of participants had completed high school or higher education. The average household size was 2.4 individuals (SD = 1.14), and >64 % were married and employed.

**Table 1:** Sample characteristics and bivariate analysis (Total *N* = 7243).

	Full sample (*N* = 7243)	Women (*N* = 3927)			Men (*N* = 3316)		
		Immigrants	Non-immigrants		Immigrants	Non-immigrants	
	M (SD) / N (%)	M (SD) / N (%)	M (SD) / N (%)	χ^2^ / *t-*test	M (SD) /N (%)	M (SD) / N (%)	χ^2^ / *t-*test
Mental health (0–100)							
2012	51.14 (9.71)	49.30 (9.36)	50.28 (10.15)	*t* = 1.91	51.42 (9.32)	52.33 (9.12)	*t* = 1.52
2014	51.99 (9.51)	49.81 (9.93)	51.16 (9.84)	*t* = 2.47*	52.49 (9.18)	53.18 (8.93)	*t* = 1.16
2016	52.68 (9.54)	50.12 (10.3)	51.90 (9.86)	*t* = 3.16**	53.18 (8.97)	53.84 (8.96)	*t* = 1.15
2018	51.83 (9.86)	49.44 (10.17)	51.24 (10.21)	*t* = 3.23**	51.41 (9.59)	52.83 (9.31)	*t* = 2.27*
2020	51.21 (9.93)	48.65 (10.45)	50.53 (10.07)	*t* = 3.26**	50.90 (10.17)	52.41 (9.53)	*t* = 2.28*
Physical health (0–100)							
2012	49.18 (9.75)	49.14 (9.41)	48.64 (10.19)	*t* = −0.97	49.53 (9.26)	49.77 (9.27)	*t* = 0.40
2014	48.75 (9.96)	47.98 (10.15)	48.31 (10.30)	*t* = 0.58	48.86 (9.81)	49.34 (9.52)	*t* = 0.76
2016	47.86 (10.00)	47.66 (10.15)	47.32 (10.45)	*t* = −0.60	48.26 (9.84)	48.48 (9.41)	*t* = 0.34
2018	47.13 (10.22)	46.76 (10.10)	46.62 (10.60)	*t* = −0.25	48.13 (9.86)	47.69 (9.78)	*t* = −0.68
2020	47.15 (10.26)	46.94 (10.20)	46.89 (10.51)	*t* = −0.09	47.80 (10.08)	47.52 (10.00)	*t* = 1.09
Loneliness in 2013 (1–5)	1.96 (0.72)	2.14 (0.78)	1.97 (0.73)	*t* = −4.01***	2.00 (0.82)	1.92 (0.67)	*t* = −1.75
Migration status							
Immigrant	634 (8.75 %)	372 (9.47 %)	-		262 (7.90 %)	-	
Non-immigrant	6609 (91.25 %)	-	3555 (90.53 %)		-	3054 (92.10 %)	
Age at migration							
18 and over	396 (68.16 %)	234 (69.23 %)	-		162 (66.67 %)	-	
0–17	185 (31.84 %)	104 (30.77 %)	-		81 (33.33 %)	-	
Age (18–80)	53.19 (14.65)	49.34 (13.31)	52.94 (14.63)	*t* = 4.92***	50.98 (14.56)	54.15 (14.72)	*t* = 3.38***
Educational attainment							
Less than high school	670 (9.25 %)	90 (24.19 %)	395 (11.11 %)	χ^2^ = 62.18***	53 (20.23 %)	132 (4.32 %)	χ^2^ = 118.08***
High school	4422 (61.05 %)	178 (47.85 %)	2278 (64.08 %)		142 (54.20 %)	1824 (59.72 %)	
Above high school	2151 (29.70 %)	104 (27.96 %)	882 (24.81 %)		67 (25.57 %)	1098 (35.95 %)	
Household income quartiles							
Lowest 25 %	1740 (24.01 %)	60 (16.13 %)	943 (26.53 %)	χ^2^ = 32.35***	51 (19.47 %)	686 (22.46 %)	χ^2^ = 10.32*
25 %−50 %	1846 (25.49 %)	122 (32.80 %)	916 (25.77 %)		78 (29.77 %)	730 (23.90 %)	
50 %−75 %	1839 (25.39 %)	117 (31.45 %)	835 (23.49 %)		80 (30.53 %)	807 (26.42 %)	
Highest 25 %	1818 (25.10 %)	73 (19.62 %)	861 (24.22 %)		53 (20.23 %)	831 (27.21 %)	
Household size (1–10)	2.40 (1.14)	2.78 (1.34)	2.34 (1.10)	*t* = −6.07***	2.77 (1.14)	2.40 (1.11)	*t* = −4.19***
Marital status							
Married	4650 (64.20 %)	271 (72.85 %)	2146 (60.37 %)	χ^2^ = 22.18***	183 (69.85 %)	2050 (67.13 %)	χ^2^ = 0.81
Others	2593 (35.80 %)	101 (27.15 %)	1409 (39.63 %)		79 (30.15 %)	1004 (32.87 %)	
Employment status							
Employed	4666 (64.42 %)	244 (65.59 %)	2188 (61.55 %)	χ^2^ = 2.34	177 (67.56 %)	2057 (67.35 %)	χ^2^ = 0
Unemployed	2577 (35.58 %)	128 (34.41 %)	1367 (38.45 %)		85 (32.44 %)	997 (32.65 %)	

**Note:***M* = mean, SD = standard deviation, *N* = count, % = percentage; *t-*test = Welch *t*-test; ***p < 0.001, **p < 0.01, *p < 0.05.

Bivariate analysis showed that non-immigrant women had better mental health than immigrant women, with the gap by migration status increasing over time (2014: *t* = 2.47, *p* < 0.05; 2016: *t* = 3.16, *p* < 0.01; 2018: *t* = 3.23, *p* < 0.01; 2020: *t* = 3.26, *p* < 0.01). Additionally, non-immigrant women reported lower loneliness levels (*t* = −4.01, *p* < 0.001), were older (*t* = 4.92, *p* < 0.001), had higher education (χ^2^ = 62.18, *p* < 0.001) and household income levels (χ^2^ = 32.35, *p* < 0.001), had smaller household sizes (*t* = −6.07, *p* < 0.001), and were less likely to be married (χ^2^ = 22.18, *p* < 0.001) than immigrant women. A similar pattern was observed among men, with non-immigrant men having better mental health (2018: *t* = 2.27, *p* < 0.05; 2020: *t* = 2.28, *p* < 0.05), being older (*t* = 3.38, *p* < 0.001), having higher education (χ^2^ = 118.08, *p* < 0.001), and household income levels (χ^2^ = 10.32, *p* < 0.05), as well as smaller household sizes (*t* = −4.19, *p* < 0.001) than immigrant men. In sum, immigrant women reported the highest levels of loneliness and the poorest mental health, compared to both non-immigrants and immigrant men.

### 4.2 PPM with mediation analysis: Migration status, loneliness, and health trajectories

**Fig. 3:**
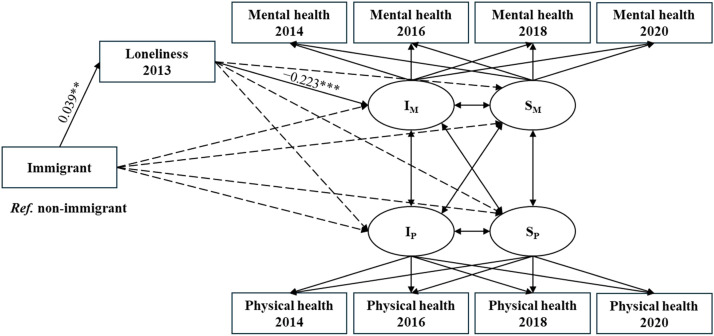
Standardized results from the mediation analysis in the parallel process latent growth curve model (*N* = 7243). **Note:** I_M_ = Intercept of mental health; S_M_ = Slope of mental health; I_P_ = Intercept of physical health; S_P_ = Slope of physical health; ***p < 0.001, **p < 0.01. The dotted lines indicate non-significant model paths. The solid lines indicate significant paths. *Ref.* = reference. Covariates: age, gender, educational attainment, household income, household size, marital status, employment status, mental and physical health in 2012, and an attrition variable to account for data loss from 2014 to 2020.

The results of the unconditional LGCM for dual health trajectories showed significant linear change for both mental and physical health, with mental and physical health declining over time (see Appendix Table A1). Fig. 3 presents the conditional PPM results with mediation analysis for the associations of migration status (reference = non-immigrant) with mental and physical health trajectories and their indirect associations through loneliness. The model showed satisfactory model fits (χ^2^(*df*) = 1225.820 (74), *p* < 0.001; CFI = 0.967; TLI = 0.936; RSMEA = 0.047; SRMR = 0.018). Results for the full sample showed that immigrants had higher loneliness levels (*β =* 0.039, *p* < 0.01), which in turn was associated with lower mental health levels (*β* = −0.223, *p* < 0.001). Table 2 presents the mediation test results, showing a significant association from migration status to loneliness, and in turn to mental health (*β =* −0.009, *p* < 0.01). However, migration status was not significantly associated with the rate of change in mental or physical health.

**Table 2:** Standardized results for the mediation analysis in the parallel process latent growth curve model.

	Full sample		Women		Men	
Model paths	*β*	*95* % *CI*	*β*	*95* % *CI*	*β*	*95* % *CI*
**Migration status (*N* = 7243)**						
Immigrant → Loneliness → I_M_	−0.009**	−0.014 - −0.003	−0.013**	−0.022 - −0.006	−0.003	−0.011 - 0.005
Immigrant → Loneliness → S_M_	0.001	−0.001 - 0.003	0.002	−0.001 - 0.007	0	−0.002 - 0.002
Immigrant → Loneliness → I_P_	0	−0.001 - 0.001	−0.001	−0.003 - 0.000	0	0.000 - 0.002
Immigrant → Loneliness → S_P_	−0.001	−0.004 - 0.001	−0.002	−0.007 - 0.002	0	−0.004 - 0.001
**Age at migration (*N* = 7190)**						
Age at migration (older than 18) → Loneliness → I_M_	−0.011***	−0.017 - −0.006	−0.015***	−0.024 - −0.008	−0.005	−0.014 - 0.003
Age at migration (older than 18) → Loneliness → S_M_	0.001	−0.001 - 0.005	0.002	−0.002 - 0.007	0	−0.002 - 0.004
Age at migration (older than 18) → Loneliness → I_P_	0	−0.002 - 0.001	−0.001	−0.004 - 0.000	0	0.000 - 0.002
Age at migration (older than 18) → Loneliness → S_P_	−0.002	−0.005 - 0.001	−0.002	−0.007 - 0.002	−0.001	−0.007 - 0.001
Age at migration (0–17) → Loneliness → I_M_	−0.001	−0.006 - 0.004	−0.004	−0.013 - 0.004	0.003	−0.002 - 0.010
Age at migration (0–17) → Loneliness → S_M_	0	0.000 - 0.002	0.001	−0.001 - 0.004	0	−0.003 - 0.001
Age at migration (0–17) → Loneliness → I_P_	0	−0.001 - 0.000	0	−0.002 - 0.000	0	−0.002 - 0.000
Age at migration (0–17) → Loneliness → S_P_	0	−0.002 - 0.000	−0.001	−0.004 - 0.000	0.001	−0.001 - 0.004

**Note:** I_M_ = Intercept of mental health; S_M_ = Slope of mental health; I_P_ = Intercept of physical health; S_P_ = Slope of physical health; β = Standardized coefficient; 95 % CI = 95 % confidence interval; ***p < 0.001, **p < 0.01.

**Fig. 4:**
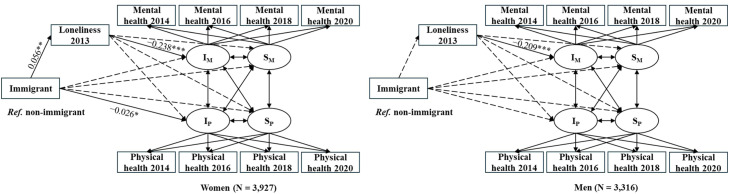
Standardized results from the mediation analysis in the parallel process latent growth curve model by gender. **Note:** I_M_ = Intercept of mental health; S_M_ = Slope of mental health; I_P_ = Intercept of physical health; S_P_ = Slope of physical health; ***p < 0.001, **p < 0.01, *p < 0.05. The dotted lines indicate non-significant model paths. The solid lines indicate significant paths. *Ref.* = reference. Covariates: age, educational attainment, household income, household size, marital status, employment status, mental and physical health in 2012, and an attrition variable to account for data loss from 2014 to 2020.

Fig. 4 and Table 2 present the findings for the subgroup analysis. It shows that this pattern was only significant among women (Immigrants – loneliness: *β =* 0.056, *p* < 0.01; loneliness – mental health: *β =* −0.238, *p* < 0.001; Immigrants – loneliness – mental health: *β =* −0.013, *p* < 0.01). Additionally, immigrant women had worse physical health than non-immigrant women (*β =* −0.026, *p* < 0.05). Among men, loneliness was significantly associated with mental health (β = −0.209, *p* < 0.001), but there was no significant link between immigrant status and loneliness, and no mediation association from immigrant status to health through loneliness (see Appendix Table A2 for detailed coefficient estimates). Overall, loneliness was found to mediate the association between migration status and mental health, but only among women.

### 4.3 PPM with mediation analysis: Age at migration, loneliness, and health trajectories

**Fig. 5:**
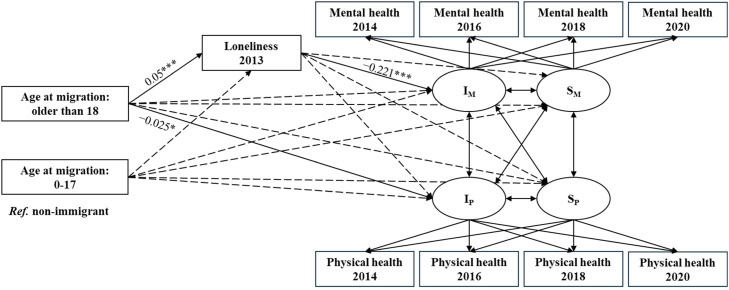
Standardized results from the mediation analysis in the parallel process latent growth curve model for the full sample (*N* = 7190). **Note:** I_M_ = Intercept of mental health; S_M_ = Slope of mental health; I_P_ = Intercept of physical health; S_P_ = Slope of physical health; ***p < 0.001, *p < 0.05. The solid lines indicate significant paths. The dotted lines indicate non-significant model paths. Non-significant paths and observed variables in the parallel process model are omitted from this figure. *Ref.* = reference. Covariates: age, educational attainment, household income, household size, marital status, employment status, mental and physical health in 2012, and an attrition variable to account for data loss from 2014 to 2020.

Fig. 5 presents the conditional PPM results for the associations of age at migration (reference = non-immigrant) with mental and physical health trajectories and their indirect associations through loneliness. The model fits are acceptable (χ^2^(*df*) = 1231.827 (74); CFI = 0.967; TLI = 0.936; RSMEA = 0.047; SRMR = 0.018). Results for the full sample showed that migrating to Germany after age 18 was associated with higher loneliness levels (*β =* 0.05, *p* < 0.001), and in turn with lower mental health levels (*β =* −0.221, *p* < 0.001). Table 2 shows a significant association from age at migration (over 18) to mental health via loneliness (*β =* −0.011, *p* < 0.001). Additionally, migrating to Germany after age 18 was associated with worse physical health (*β =* −0.025, *p* < 0.05).

**Fig. 6:**
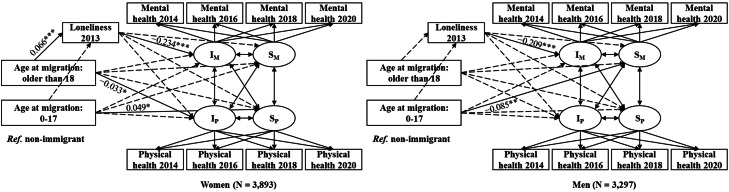
Standardized results from the mediation analysis in the parallel process latent growth curve model by gender. **Note:** I_M_ = Intercept of mental health; S_M_ = Slope of mental health; I_P_ = Intercept of physical health; S_P_ = Slope of physical health; ***p < 0.001, **p < 0.01, *p < 0.05. The solid lines indicate significant paths. The dotted lines indicate non-significant model paths. Non-significant paths and observed variables in the parallel process model are omitted from this figure. *Ref.* = reference. Covariates: age, educational attainment, household income, household size, marital status, employment status, mental and physical health in 2012, and an attrition variable to account for data loss from 2014 to 2020.

The subgroup analysis indicates that the link through loneliness was only significant among women. Specifically, for women, migrating after age 18 was associated with increased loneliness (*β* = 0.066, *p* < 0.001), and in turn with poorer mental health (*β* = −0.234, *p* < 0.001), resulting in an overall significant indirect association (*β* = −0.015, *p* < 0.001; see Table 2 and Fig. 6). Immigrant women who migrated after age 18 had worse physical health than non-immigrant women (*β* = −0.033, *p* < 0.05). For men, we found no association between immigrant status and health through loneliness. Men who migrated at younger ages (0–17) experienced faster mental health decline than non-immigrant men (*β* = −0.085, *p* < 0.01) (see Appendix Table A3 for detailed coefficient estimates). The mediating role of loneliness in the link between migration status and mental health was observed only among immigrant women who migrated after age 18.

### 4.4 Sensitivity test

To ensure the robustness of our results, we conducted three sensitivity tests. First, we accounted for immigrants’ length of stay, categorizing them into two groups: those who had stayed for <10 years and those who had stayed for >10 years. However, the statistical power was reduced due to the limited sample size of the former group (immigrant men who had stayed for <10 years: 27). The results remained consistent, showing that both short- and long-term immigrant women were more likely to experience loneliness, which was associated with worse mental health (see Appendix Figures A1 and A2). Second, we reanalyzed the model to account for potential COVID-19 effects by excluding the 2020 wave. Although the model resulted in an inadmissible solution (i.e., negative variances of growth parameters), the findings remained consistent, showing that the mediation path from migration status to mental health through loneliness persisted (see Appendix Figures A3 and A4). Finally, we divide age at migration into four groups, distinguishing those who moved to Germany at ages 0–5, 6–17, 18–34 and 35+, following previous studies (Gubernskaya, 2014). Consistent with our main results, immigrant women who migrated to Germany at ages 18–34 or 35+ are more likely to experience loneliness, which is associated with their mental health (see Figures A5 and A6).

## 5 Discussion

This study explores how loneliness contributes to mental and physical health disparities between immigrants and non-immigrants, and whether these relationships differ by gender and age at migration. Moreover, this study introduces an innovative approach that can directly examine the mechanisms underlying health disparities across migration status. The descriptive and bivariate analyses reveal that immigrants reported worse mental health than non-immigrants. This gap is particularly pronounced among women which in line with previous studies (Loi et al., 2025; Potochnick and Perreira, 2010). We find no evidence for the healthy immigrant effect on mental and physical health, as our observation period starts in 2012, with most immigrants in our sample (92.25 %) having lived in Germany for over 10 years. This is consistent with previous research, which shows that the healthy immigrant effect disappears after a length of stay of 10 years or more (Loi and Hale, 2019). Additionally, immigrants report higher levels of loneliness than non-immigrants, with immigrant women reporting the highest levels of loneliness than other groups, consistent with previous studies (De Witte and Van Regenmortel, 2022; Delaruelle, 2023; Fokkema and Naderi, 2013). The findings provide empirical evidence from Germany that immigrant women face higher mental health risks and weaker integration into personal networks, as reflected in higher levels of loneliness.

The PPM with mediation analysis suggests an indirect association between migration status and mental health (i.e., intercept) through loneliness, but not with the rate of change in mental health (i.e., slope), thereby partially supporting H1.1. The findings are consistent with previous studies linking immigrants to loneliness and loneliness to health outcomes, while extending this research by showing a mediation path from migration status to health outcomes via loneliness (Hawkley, 2022; Joshi et al., 2024; Wu and Penning, 2015). These findings highlight the importance of social integration for immigrants’ mental health (Delaruelle, 2023). A lack of integration into personal networks, reflected in higher loneliness, contributes to mental health disparities between immigrants and non-immigrants. These findings further highlight the role of socio-psychological factors in explaining health disparities by migration status, an area that has received limited attention in prior research.

However, we do not find an association between migration status and the rate of mental health change through loneliness. This aligns with Brunori (2024), who found no evidence that immigrants experience faster mental health decline than non-immigrants. This pattern may be due to the fact that most immigrants in the sample have been in the country for a long time. As immigrants already have lower mental health, leaving less room for further deterioration compared with non-immigrants. Therefore, we find no difference in the rate of mental health change between immigrants and non-immigrants. Moreover, no significant mediation via loneliness was observed for physical health, which may be because physical health is largely explained by mental health in our model. We examined the interrelated trajectories of mental and physical health simultaneously, and loneliness may affect physical health indirectly through mental health. This may explain why no significant direct link is observed between loneliness and physical health.

The subgroup analysis by gender shows that the mediation path is only significant among women, supporting H1.2. Specifically, the association between migration status and loneliness is significant only among immigrant women. Immigrant women are more likely to experience loneliness, which is in turn associated with poorer mental health. This findings echo previous studies, highlighting that immigrant women are particularly vulnerable and that the factors contributing to health disparities among immigrants and non-immigrants may vary by gender (Llácer et al., 2007).

Loneliness contributes to mental health disparities across migration status among women for several reasons. First, evidence shows that immigrant women face additional challenges in rebuilding their social networks in the receiving country, which can lead to increased loneliness (Hurtado-de-Mendoza et al., 2014). This suggests that immigrant women experience greater difficulty achieving social integration compared to immigrant men, leading to higher levels of loneliness and poorer mental health. In addition, evidence shows that women tend to suffer more from a lack of social support compared with men (Shin and Park, 2023), and men and women may evaluate loneliness differently (Joshi et al., 2024). These factors may contribute to higher reports of loneliness among immigrant women compared with men. However, these explanations require further examination in future research.

The results from the PPM mediation analysis indicate an indirect association between migration status and mental health through loneliness, specifically for individuals who migrated to Germany after age 18. Individuals who migrated at older ages are more likely to experience loneliness compared to non-immigrants, which contributes to poor mental health. Moreover, these individuals have worse physical health than non-immigrants. Thus, H2.1 is partially supported. These findings align with previous studies suggesting that individuals who migrate at older ages are particularly vulnerable, as they face greater challenges integrating into the receiving country (Delaruelle, 2023; Honkaniemi et al., 2020). The findings indicate the importance of the socialization process in the receiving country, as migrating later in life can hinder adaptation and inclusion. For example, older immigrants are more likely than younger ones to encounter language and cultural barriers, which are associated with higher loneliness and poorer mental health (Treas and Batalova, 2009; Treas and Mazumdar, 2002).

The subgroup analysis by gender reveals a similar pattern for migration status, showing that the indirect association between migration status and mental health via loneliness is only significant among women. Thus, H2.2 is supported. The findings indicate that immigrant women who migrated after age 18 are particularly vulnerable, as they are more likely to experience loneliness, which undermines their mental health. These results echo Delaruelle (2023), who finds that women and immigrants who migrate at older ages are more vulnerable to loneliness. The findings highlight the importance of considering both gender and migration-related factors in research on migrant health.

The limitations of this study should be noted. First, because data on loneliness were collected from 2013, our analysis captures health disparities between immigrants and non-immigrants within a specific time window, rather than tracking health trajectories since their migration. Therefore, we cannot directly test the healthy immigrant effect or assimilation hypothesis, but can only show health disparities and their changes at specific time points. Future studies with more comprehensive data could adopt a similar research design but track immigrants’ health changes from arrival to older ages and compare them with those of non-immigrants. This would allow a direct examination of how loneliness interacts with the healthy immigrant effect or the unhealthy assimilation hypothesis.

Second, loneliness was measured using the UCLA Loneliness Scale, which captures emotional loneliness rather than objective social ties. As a self-reported measure, it may be subject to reporting bias and may vary across cultural backgrounds. Nevertheless, we found that immigrants report higher levels of loneliness than non-immigrants. Future research could include measures of social ties to better understand the underlying mechanisms.

Third, although PCS and MCS have good reliability and validity (Ware et al., 2002), these health outcomes are self-reported, which may also introduce recall bias. Future studies could replicate this study design to examine health disparities using more objective mental and physical health indicators. Third, this study only investigates the mechanisms in the German context. Although our findings are generally consistent with previous literature and Germany is a key receiving country in Europe, the underlying mechanisms may vary across contexts and require further investigation.

Fourth, although we use a longitudinal design, the observed associations should not be interpreted as causal. Future studies could apply advanced causal methods to better identify causal relationships and uncover more detailed underlying mechanisms. Additionally, despite the fact that G-SOEP is Germany’s largest and longest-running longitudinal dataset with an oversampling of immigrants that is well suited to this study, it has an attrition rate of around nine percent (Giesselmann et al., 2019).

Finally, even though we removed respondents over age 80, we cannot fully control for the “salmon bias” effect (Palloni and Arias, 2004). However, in our study, we find that older immigrants have worse health outcomes than non-immigrants. This implies that if we could fully account for selective out-migration, the observed health disparities would likely be even larger. Therefore, we are confident in the robustness and conservative nature of our findings.

Despite these limitations, this study makes theoretical, methodological, and empirical contributions. Theoretically, this study advances research on behavioral and social structural explanations of health disparities by migration status. It highlights loneliness as an important mechanism in explaining health disparities between immigrants and non-immigrants. It also highlights the need for future research to investigate additional socio-psychological mechanisms, such as relative deprivation, perceived discrimination, and sense of belonging, in shaping migrant health disparities. Furthermore, the observed differences in mechanisms by gender and age at migration underscore the need for research frameworks that explicitly consider these factors to better understand the intersections of gender, migration, and health.

Methodologically, compared to conventional random or fixed regression models, the PPM approach with mediation analysis has several advantages. First, the PPM can handle measurement errors with latent constructs (Muthen, 2004). Second, it allows for the inclusion of multiple outcomes in a single model, enabling the measurement of correlations between outcomes, as different dimensions of health are often correlated. Third, it distinguishes between individual differences and within-individual changes, helping to clarify how health trajectories vary across migration status (Wickrama et al., 2016). Finally, it is flexible and can directly examine the mechanisms for various research purposes, such as incorporating mediating factors to investigate underlying processes, which can generate clearer and more direct results (Cheong et al., 2003).

Empirically, our findings show that women who migrated to Germany after age 18 are particularly vulnerable to loneliness, which is associated with poorer mental health. Policies aimed at improving immigrants’ mental health should prioritize immigrant women who migrated after age 18, as they appear to be particularly vulnerable to loneliness and poor mental health outcomes. Targeted interventions should focus on reducing loneliness and strengthening social integration for this group to facilitate their adjustment in the receiving country.

## Funding

This work was supported by the 10.13039/501100003529European Union (ERC Starting Grant, MigHealthGaps, 101116721). Views and opinions expressed are however those of the authors only and do not necessarily reflect those of the European Union or the European Research Council. Neither the European Union nor the granting authority can be held responsible for them.

## CRediT authorship contribution statement

**Songyun Shi:** Writing – review & editing, Writing – original draft, Visualization, Validation, Methodology, Formal analysis, Data curation, Conceptualization. **Silvia Loi:** Writing – review & editing, Visualization, Validation, Supervision, Funding acquisition, Conceptualization.

## Declaration of competing interest

The authors have no conflicts of interest relevant to this article.

## Appendix Supplementary materials

Image, application 1
