# Supplementary material for: How loneliness mediates the association between migration status and health trajectories: Longitudinal evidence from Germany
Source: J Migr Health. 2026 Mar 30;13:100408. doi: 10.1016/j.jmh.2026.100408 (PMC13091140; doi:10.1016/j.jmh.2026.100408)
Supplement: Supplementary file 1 [file mmc1.docx]

**Table A1.** Unstandardized estimates for unconditional latent growth curve models for full samples and subsamples

|  | **Mean** | | | **Variance** | | |
| --- | --- | --- | --- | --- | --- | --- |
|  | **Estimate** | **SE** | ***p*** | **Estimate** | **SE** | ***p*** |
| Full sample |  |  |  |  |  |  |
| I_M_ | 52.353 | 0.106 | 0.000 | 51.606 | 1.519 | 0.000 |
| S_M_ | −0.296 | 0.042 | 0.000 | 3.264 | 0.288 | 0.000 |
| I_P_ | 48.596 | 0.112 | 0.000 | 69.251 | 1.603 | 0.000 |
| S_P_ | −0.624 | 0.035 | 0.000 | 2.201 | 0.207 | 0.000 |
| Women |  |  |  |  |  |  |
| I_M_ | 51.399 | 0.150 | 0.000 | 55.409 | 2.244 | 0.000 |
| S_M_ | −0.253 | 0.060 | 0.000 | 3.757 | 0.430 | 0.000 |
| I_P_ | 48.075 | 0.159 | 0.000 | 76.263 | 2.345 | 0.000 |
| S_P_ | −0.562 | 0.049 | 0.000 | 2.536 | 0.296 | 0.000 |
| Men |  |  |  |  |  |  |
| I_M_ | 53.485 | 0.147 | 0.000 | 44.911 | 1.959 | 0.000 |
| S_M_ | −0.348 | 0.058 | 0.000 | 2.698 | 0.375 | 0.000 |
| I_P_ | 49.218 | 0.157 | 0.000 | 60.089 | 2.132 | 0.000 |
| S_P_ | −0.697 | 0.050 | 0.000 | 1.783 | 0.287 | 0.000 |

**Note.** I_M_ = Intercept of mental health; S_M_ = Slope of mental health; I_P_ = Intercept of physical health; S_P_ = Slope of physical health.

**Table A2.** Standardized results for mediation effects from the mediation analysis in the parallel process latent growth curve model of migration background (Total *N* = 7,243)

|  | **Full sample (*N* = 7,243)** | | **Women (*N* = 3,927)** | | **Men (*N* = 3,316)** | |
| --- | --- | --- | --- | --- | --- | --- |
|  | ***β*** | ***SE*** | ***β*** | ***SE*** | ***β*** | ***SE*** |
| Immigrant → I_M_ | −0.005 | 0.011 | −0.014 | 0.015 | 0.009 | 0.017 |
| Immigrant → S_M_ | −0.04 | 0.022 | −0.028 | 0.029 | −0.057 | 0.037 |
| Immigrant → I_P_ | −0.018 | 0.009 | −0.026* | 0.013 | −0.004 | 0.014 |
| Immigrant → S_P_ | 0.021 | 0.021 | 0.034 | 0.029 | 0.001 | 0.036 |
| Immigrant → Loneliness | 0.039** | 0.012 | 0.056** | 0.016 | 0.013 | 0.02 |
| Loneliness → I_M_ | −0.223*** | 0.015 | −0.238*** | 0.019 | −0.209*** | 0.021 |
| Loneliness → S_M_ | 0.023 | 0.025 | 0.037 | 0.033 | 0.001 | 0.04 |
| Loneliness → I_P_ | −0.006 | 0.01 | −0.02 | 0.014 | 0.014 | 0.014 |
| Loneliness → S_P_ | −0.036 | 0.024 | −0.035 | 0.035 | −0.033 | 0.04 |
| Age → I_M_ | 0.122*** | 0.017 | 0.103*** | 0.023 | 0.152*** | 0.025 |
| Age → S_M_ | 0.048 | 0.032 | 0.058 | 0.042 | 0.027 | 0.048 |
| Age → I_P_ | −0.165*** | 0.013 | −0.159*** | 0.018 | −0.176*** | 0.018 |
| Age → S_P_ | −0.157*** | 0.031 | −0.073 | 0.041 | −0.275*** | 0.049 |
| Age → Loneliness | −0.142*** | 0.016 | −0.165*** | 0.019 | −0.095*** | 0.024 |
| Men → I_M_ | 0.058*** | 0.012 |  |  |  |  |
| Men → S_M_ | −0.005 | 0.022 |  |  |  |  |
| Men → I_P_ | 0.012 | 0.009 |  |  |  |  |
| Men → S_P_ | −0.031 | 0.022 |  |  |  |  |
| Men → Loneliness | 0.021 | 0.011 |  |  |  |  |
| Education → I_M_ | 0.015 | 0.012 | 0.025 | 0.016 | 0.001 | 0.02 |
| Education → S_M_ | −0.034 | 0.024 | −0.031 | 0.031 | −0.035 | 0.04 |
| Education → I_P_ | 0.066* | 0.01 | 0.051*** | 0.013 | 0.085*** | 0.015 |
| Education → S_P_ | 0.045 | 0.024 | 0.042 | 0.029 | 0.069 | 0.037 |
| Education → Loneliness | −0.022 | 0.012 | −0.008 | 0.016 | −0.049** | 0.017 |
| Household income → I_M_ | −0.023 | 0.017 | −0.031 | 0.025 | −0.013 | 0.025 |
| Household income → S_M_ | 0.083* | 0.033 | 0.048 | 0.044 | 0.131* | 0.053 |
| Household income → I_P_ | 0.036** | 0.014 | 0.049** | 0.019 | 0.021 | 0.02 |
| Household income → S_P_ | 0.033 | 0.033 | 0.055 | 0.044 | 0.005 | 0.052 |
| Household income → Loneliness | −0.066*** | 0.016 | −0.065** | 0.022 | −0.073** | 0.024 |
| Household size → I_M_ | −0.025 | 0.016 | −0.03 | 0.022 | −0.021 | 0.021 |
| Household size → S_M_ | 0.004 | 0.027 | 0.026 | 0.038 | −0.026 | 0.041 |
| Household size → I_P_ | 0.002 | 0.012 | 0.005 | 0.018 | 0 | 0.016 |
| Household size → S_P_ | −0.023 | 0.027 | −0.042 | 0.038 | 0.008 | 0.042 |
| Household size → Loneliness | −0.024 | 0.014 | −0.019 | 0.019 | −0.028 | 0.021 |
| Married → I_M_ | 0.002 | 0.015 | 0.007 | 0.02 | −0.005 | 0.022 |
| Married → S_M_ | −0.034 | 0.028 | −0.041 | 0.035 | −0.014 | 0.045 |
| Married → I_P_ | −0.006 | 0.011 | −0.009 | 0.015 | −0.004 | 0.017 |
| Married → S_P_ | 0.05 | 0.028 | 0.082* | 0.036 | 0.011 | 0.045 |
| Married → Loneliness | −0.049*** | 0.013 | −0.03 | 0.017 | −0.082*** | 0.021 |
| Employed → I_M_ | 0.021 | 0.016 | 0.013 | 0.022 | 0.032 | 0.027 |
| Employed → S_M_ | 0.031 | 0.033 | 0.045 | 0.041 | 0.004 | 0.053 |
| Employed → I_P_ | −0.007 | 0.013 | −0.009 | 0.018 | −0.002 | 0.02 |
| Employed → S_P_ | 0.017 | 0.032 | 0.04 | 0.042 | −0.024 | 0.053 |
| Employed → Loneliness | −0.008 | 0.017 | −0.014 | 0.022 | 0.01 | 0.026 |
| Mental health in 2012 → I_M_ | 0.584*** | 0.015 | 0.572*** | 0.02 | 0.607*** | 0.02 |
| Mental health in 2012 → S_M_ | −0.192*** | 0.028 | −0.179*** | 0.037 | −0.207*** | 0.045 |
| Mental health in 2012 → I_P_ | 0.116*** | 0.011 | 0.105*** | 0.014 | 0.13*** | 0.017 |
| Mental health in 2012 → S_P_ | −0.022 | 0.024 | −0.02 | 0.032 | −0.023 | 0.043 |
| Mental health in 2012 → Loneliness | −0.347*** | 0.012 | −0.346*** | 0.017 | −0.345*** | 0.018 |
| Physical health in 2012 → I_M_ | 0.089*** | 0.015 | 0.093*** | 0.019 | 0.085*** | 0.022 |
| Physical health in 2012 → S_M_ | 0.047 | 0.026 | 0.036 | 0.034 | 0.066 | 0.044 |
| Physical health in 2012 → I_P_ | 0.72*** | 0.01 | 0.71*** | 0.013 | 0.736*** | 0.014 |
| Physical health in 2012 → S_P_ | −0.198*** | 0.026 | −0.185*** | 0.036 | −0.216*** | 0.045 |
| Physical health in 2012 → Loneliness | −0.161*** | 0.013 | −0.165*** | 0.017 | −0.157*** | 0.02 |
| Attrition → I_M_ | −0.016 | 0.012 | −0.011 | 0.018 | −0.022 | 0.017 |
| Attrition → S_M_ | −0.014 | 0.028 | 0.023 | 0.036 | −0.069 | 0.042 |
| Attrition → I_P_ | −0.013 | 0.01 | −0.005 | 0.013 | −0.02 | 0.014 |
| Attrition → S_P_ | −0.037 | 0.027 | −0.038 | 0.037 | −0.037 | 0.044 |
| Attrition → Loneliness | −0.004 | 0.011 | 0.02 | 0.014 | −0.034* | 0.016 |

**Note.** I_M_ = Intercept of mental health; S_M_ = Slope of mental health; I_P_ = Intercept of physical health; S_P_ = Slope of physical health; β = Standardized coefficient; SE = Standard Error; ***p < 0.001, **p < 0.01, *p < 0.05.

**Table A3.** Standardized results for mediation effects from the mediation analysis in the parallel process latent growth curve model of age at migration (Total *N* = 7,190)

|  | **Full sample (*N* = 7,190)** | | **Women (*N* = 3,893)** | | **Men (*N* = 3,297)** | |
| --- | --- | --- | --- | --- | --- | --- |
|  | ***β*** | ***SE*** | ***β*** | ***SE*** | ***β*** | ***SE*** |
| Age at migration |  |  |  |  |  |  |
| 0-17 → I_M_ | −0.003 | 0.011 | −0.018 | 0.015 | 0.017 | 0.015 |
| 0-17 → S_M_ | −0.019 | 0.021 | 0.024 | 0.028 | −0.085* | 0.032 |
| 0-17 → I_P_ | 0.009 | 0.008 | 0.012 | 0.011 | 0.007 | 0.013 |
| 0-17 → S_P_ | 0.036 | 0.019 | 0.049* | 0.024 | 0.018 | 0.03 |
| 0-17 → Loneliness | 0.003 | 0.012 | 0.017 | 0.017 | −0.016 | 0.014 |
| 18 and over → I_M_ | −0.006 | 0.012 | −0.011 | 0.015 | 0.001 | 0.018 |
| 18 and over → S_M_ | −0.035 | 0.021 | −0.047 | 0.027 | −0.013 | 0.037 |
| 18 and over → I_P_ | −0.025* | 0.01 | −0.033* | 0.013 | −0.012 | 0.016 |
| 18 and over → S_P_ | −0.002 | 0.023 | 0.002 | 0.03 | −0.006 | 0.037 |
| 18 and over → Loneliness | 0.05*** | 0.013 | 0.066*** | 0.017 | 0.025 | 0.022 |
| Loneliness → I_M_ | −0.221*** | 0.014 | −0.234*** | 0.02 | −0.209*** | 0.021 |
| Loneliness → S_M_ | 0.024 | 0.026 | 0.034 | 0.035 | 0.006 | 0.041 |
| Loneliness → I_P_ | −0.006 | 0.011 | −0.02 | 0.015 | 0.014 | 0.015 |
| Loneliness → S_P_ | −0.033 | 0.026 | −0.03 | 0.034 | −0.035 | 0.04 |
| Age → I_M_ | 0.123*** | 0.017 | 0.103*** | 0.023 | 0.156*** | 0.024 |
| Age → S_M_ | 0.044 | 0.032 | 0.059 | 0.039 | 0.012 | 0.047 |
| Age → I_P_ | −0.162*** | 0.013 | −0.155*** | 0.018 | −0.175*** | 0.019 |
| Age → S_P_ | −0.156*** | 0.031 | −0.075 | 0.041 | −0.273*** | 0.047 |
| Age → Loneliness | −0.145*** | 0.016 | −0.168*** | 0.019 | −0.099*** | 0.024 |
| Men → I_M_ | 0.058*** | 0.012 |  |  |  |  |
| Men → S_M_ | −0.006 | 0.022 |  |  |  |  |
| Men → I_P_ | 0.011 | 0.009 |  |  |  |  |
| Men → S_P_ | −0.028 | 0.022 |  |  |  |  |
| Men → Loneliness | 0.02 | 0.011 |  |  |  |  |
| Education → I_M_ | 0.016 | 0.013 | 0.027 | 0.017 | 0.002 | 0.019 |
| Education → S_M_ | −0.035 | 0.024 | −0.031 | 0.031 | −0.039 | 0.04 |
| Education → I_P_ | 0.067*** | 0.01 | 0.052*** | 0.014 | 0.086*** | 0.015 |
| Education → S_P_ | 0.041 | 0.023 | 0.037 | 0.032 | 0.066 | 0.038 |
| Education → Loneliness | −0.021 | 0.012 | −0.008 | 0.016 | −0.046** | 0.017 |
| Household income → I_M_ | −0.024 | 0.017 | −0.031 | 0.024 | −0.015 | 0.025 |
| Household income → S_M_ | 0.086** | 0.032 | 0.048 | 0.045 | 0.143** | 0.05 |
| Household income → I_P_ | 0.036** | 0.014 | 0.051** | 0.018 | 0.019 | 0.021 |
| Household income → S_P_ | 0.033 | 0.032 | 0.052 | 0.042 | 0.01 | 0.051 |
| Household income → Loneliness | −0.066*** | 0.016 | −0.063** | 0.023 | −0.075** | 0.024 |
| Household size → I_M_ | −0.024 | 0.015 | −0.028 | 0.021 | −0.02 | 0.021 |
| Household size → S_M_ | 0.003 | 0.029 | 0.025 | 0.039 | −0.029 | 0.04 |
| Household size → I_P_ | 0.002 | 0.012 | 0.005 | 0.017 | −0.001 | 0.017 |
| Household size → S_P_ | −0.022 | 0.028 | −0.042 | 0.038 | 0.009 | 0.042 |
| Household size → Loneliness | −0.026 | 0.014 | −0.022 | 0.019 | −0.028 | 0.02 |
| Married → I_M_ | 0.001 | 0.015 | 0.005 | 0.02 | −0.006 | 0.022 |
| Married → S_M_ | −0.029 | 0.028 | −0.035 | 0.037 | −0.008 | 0.044 |
| Married → I_P_ | −0.006 | 0.012 | −0.008 | 0.015 | −0.004 | 0.018 |
| Married → S_P_ | 0.05 | 0.028 | 0.082* | 0.035 | 0.01 | 0.045 |
| Married → Loneliness | −0.048** | 0.014 | −0.029 | 0.017 | −0.082*** | 0.022 |
| Employed → I_M_ | 0.022 | 0.017 | 0.012 | 0.023 | 0.038 | 0.027 |
| Employed → S_M_ | 0.024 | 0.033 | 0.043 | 0.041 | −0.011 | 0.053 |
| Employed → I_P_ | −0.008 | 0.014 | −0.011 | 0.018 | 0 | 0.02 |
| Employed → S_P_ | 0.011 | 0.032 | 0.035 | 0.042 | −0.031 | 0.052 |
| Employed → Loneliness | −0.006 | 0.016 | −0.013 | 0.022 | 0.013 | 0.025 |
| Mental health in 2012 → I_M_ | 0.584*** | 0.014 | 0.572*** | 0.02 | 0.607*** | 0.02 |
| Mental health in 2012 → S_M_ | −0.191*** | 0.028 | −0.178*** | 0.038 | −0.207*** | 0.043 |
| Mental health in 2012 → I_P_ | 0.115*** | 0.011 | 0.104*** | 0.015 | 0.13*** | 0.017 |
| Mental health in 2012 → S_P_ | −0.022 | 0.024 | −0.019 | 0.032 | −0.025 | 0.043 |
| Mental health in 2012 → Loneliness | −0.347*** | 0.012 | −0.346*** | 0.016 | −0.345*** | 0.019 |
| Physical health in 2012 → I_M_ | 0.087*** | 0.014 | 0.091*** | 0.02 | 0.084*** | 0.022 |
| Physical health in 2012 → S_M_ | 0.05 | 0.026 | 0.037 | 0.034 | 0.074 | 0.041 |
| Physical health in 2012 → I_P_ | 0.72*** | 0.01 | 0.71*** | 0.014 | 0.735*** | 0.015 |
| Physical health in 2012 → S_P_ | −0.198*** | 0.027 | −0.182*** | 0.036 | −0.219*** | 0.047 |
| Physical health in 2012 → Loneliness | −0.161*** | 0.013 | −0.164*** | 0.017 | −0.158*** | 0.02 |
| Attrition → I_M_ | −0.017 | 0.013 | −0.013 | 0.017 | −0.022 | 0.018 |
| Attrition → S_M_ | −0.012 | 0.028 | 0.026 | 0.037 | −0.068 | 0.043 |
| Attrition → I_P_ | −0.012 | 0.01 | −0.004 | 0.014 | −0.02 | 0.014 |
| Attrition → S_P_ | −0.041 | 0.029 | −0.043 | 0.036 | −0.041 | 0.042 |
| Attrition → Loneliness | −0.004 | 0.011 | 0.021 | 0.014 | −0.035*** | 0.016 |

**Note.** I_M_ = Intercept of mental health; S_M_ = Slope of mental health; I_P_ = Intercept of physical health; S_P_ = Slope of physical health; β = Standardized coefficient; SE = Standard Error; ***p < 0.001, **p < 0.01, *p < 0.05.


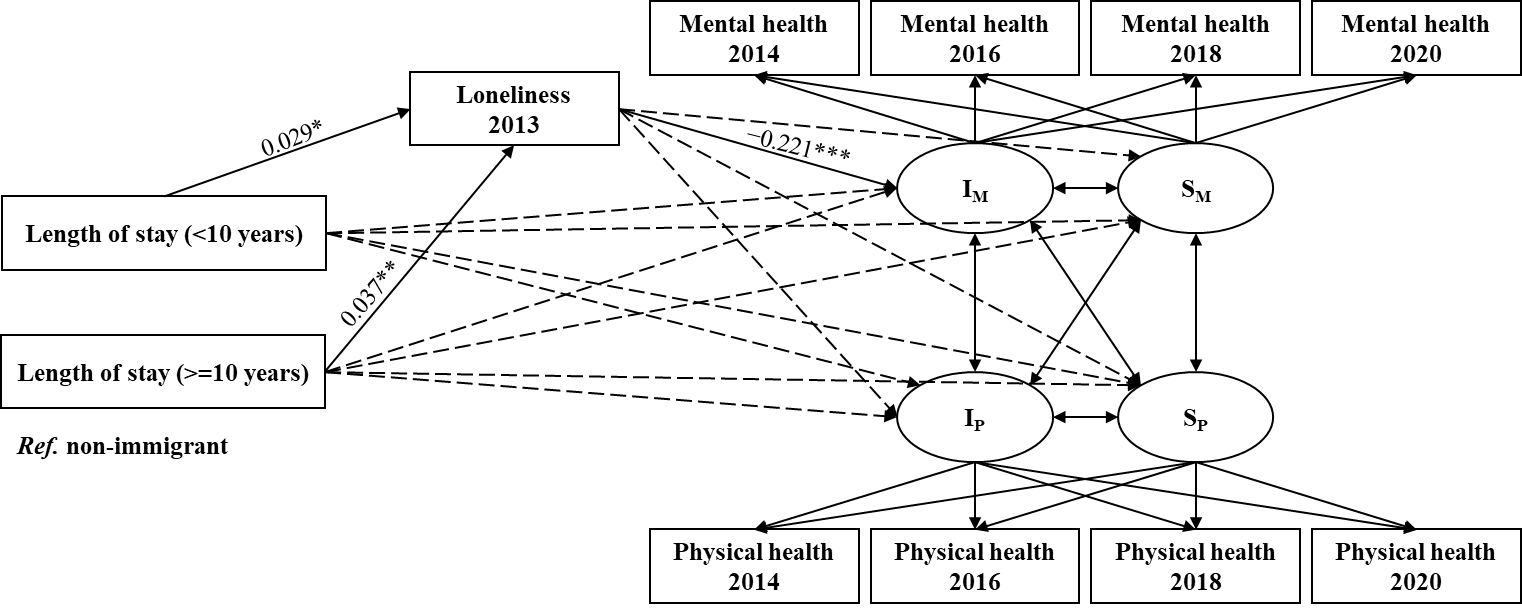


**Figure A1.** Standardized results from the mediation analysis in the parallel process latent growth curve model of length of stay (N = 7,190)

**Note.** I_M_ = Intercept of mental health; S_M_ = Slope of mental health; I_P_ = Intercept of physical health; S_P_ = Slope of physical health; The dotted lines indicate non-significant model paths; The solid lines indicate significant paths; Covariates: age, gender, educational attainment, household income, household size, marital status, employment status, mental and physical health in 2012, and an attrition variable to account for data loss from 2014 to 2020.


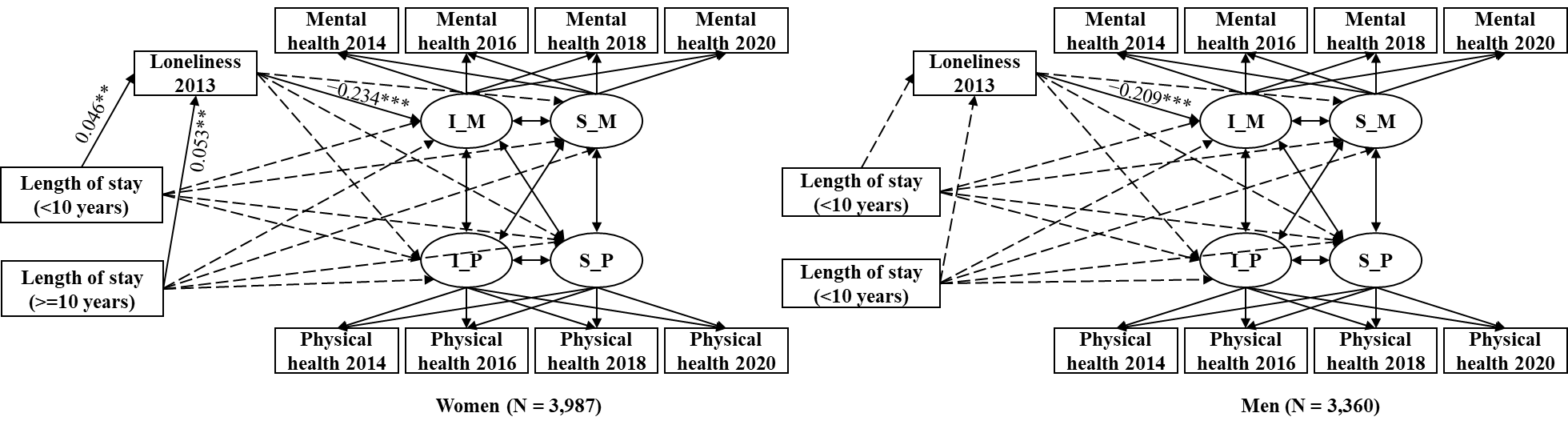


**Figure A2.** Standardized results from the mediation analysis in the parallel process latent growth curve model of length of stay by gender

**Note.** I_M_ = Intercept of mental health; S_M_ = Slope of mental health; I_P_ = Intercept of physical health; S_P_ = Slope of physical health; The dotted lines indicate non-significant model paths; The solid lines indicate significant paths; Covariates: age, educational attainment, household income, household size, marital status, employment status, mental and physical health in 2012, and an attrition variable to account for data loss from 2014 to 2020.


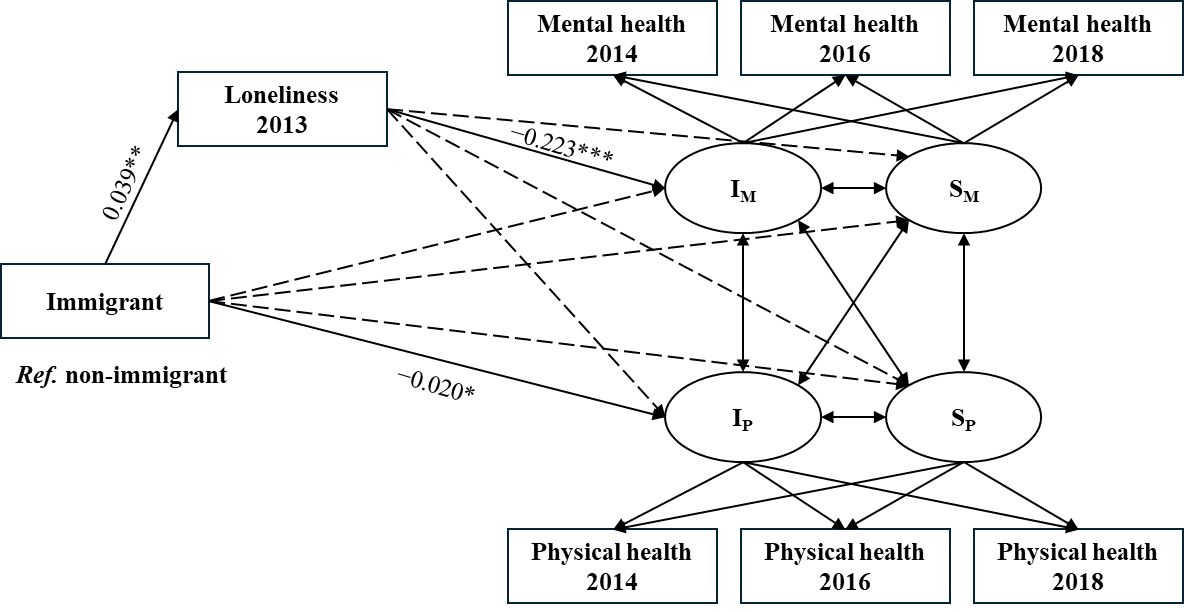


**Figure A3.** Standardized results from the mediation analysis in the parallel process latent growth curve model across three waves (N = 7,243)

**Note.** I_M_ = Intercept of mental health; S_M_ = Slope of mental health; I_P_ = Intercept of physical health; S_P_ = Slope of physical health; The dotted lines indicate non-significant model paths; The solid lines indicate significant paths; Covariates: age, gender, educational attainment, household income, household size, marital status, employment status, mental and physical health in 2012, and an attrition variable to account for data loss from 2014 to 2020.


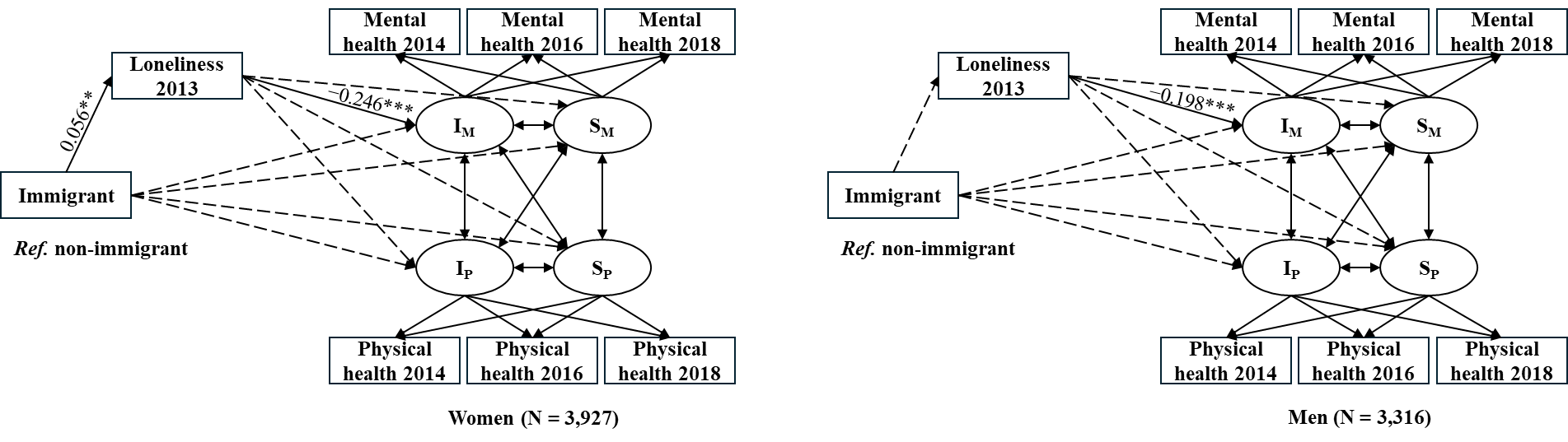


**Figure A4.** Standardized results from the mediation analysis in the parallel process latent growth curve model across three waves by gender

**Note.** I_M_ = Intercept of mental health; S_M_ = Slope of mental health; I_P_ = Intercept of physical health; S_P_ = Slope of physical health; The dotted lines indicate non-significant model paths; The solid lines indicate significant paths; Covariates: age, educational attainment, household income, household size, marital status, employment status, mental and physical health in 2012, and an attrition variable to account for data loss from 2014 to 2020.


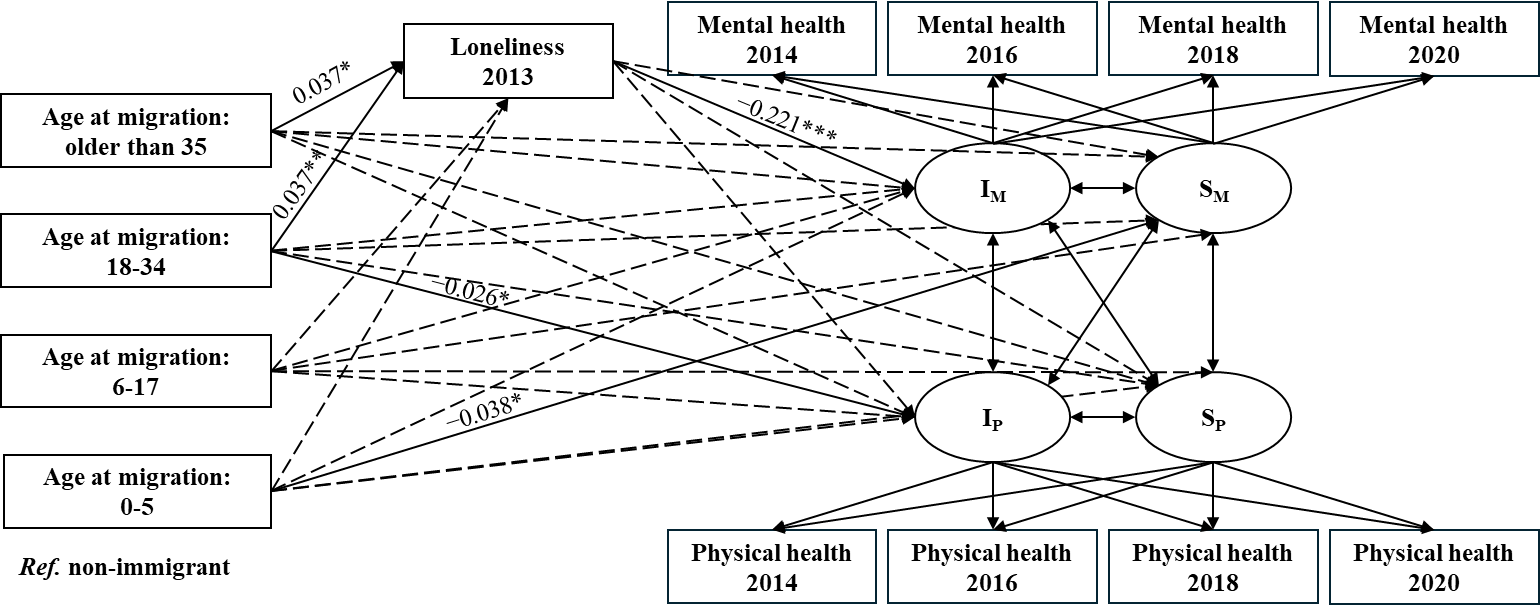


**Figure A5.** Standardized results from the mediation analysis in the parallel process latent growth curve model for age at migration (N = 7,190)

**Note.** I_M_ = Intercept of mental health; S_M_ = Slope of mental health; I_P_ = Intercept of physical health; S_P_ = Slope of physical health; The dotted lines indicate non-significant model paths; The solid lines indicate significant paths; Covariates: age, gender, educational attainment, household income, household size, marital status, employment status, mental and physical health in 2012, and an attrition variable to account for data loss from 2014 to 2020.


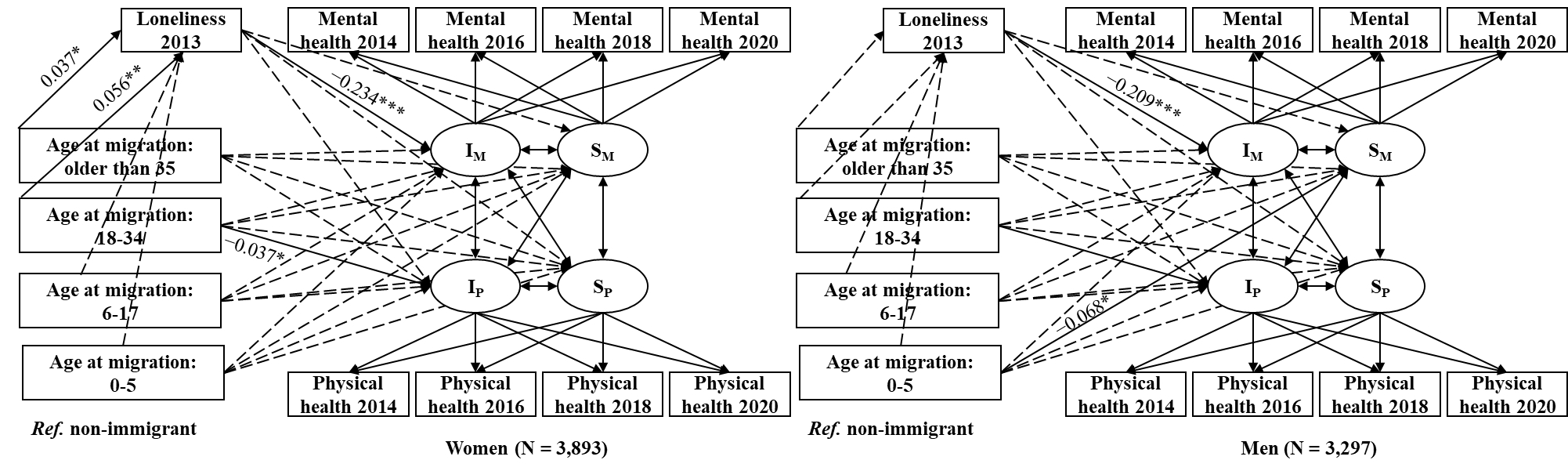


**Figure A6.** Standardized results from the mediation analysis in the parallel process latent growth curve model for age at migration by gender

**Note.** I_M_ = Intercept of mental health; S_M_ = Slope of mental health; I_P_ = Intercept of physical health; S_P_ = Slope of physical health; The dotted lines indicate non-significant model paths; The solid lines indicate significant paths; Covariates: age, educational attainment, household income, household size, marital status, employment status, mental and physical health in 2012, and an attrition variable to account for data loss from 2014 to 2020.
